# 异基因造血干细胞移植后合并吉兰-巴雷综合征1例报告并文献复习

**DOI:** 10.3760/cma.j.cn121090-20231020-00222

**Published:** 2024-05

**Authors:** 亚军 石, 英 汉, 莹 王, 芮 周, 瑞 宋, 东锋 毛, 瑞 葸, 海 白, 涛 吴

**Affiliations:** 中国人民解放军联勤保障部队第九四〇医院血液科，兰州 730050 Hematology Department, 940 Hospital of the Joint Logistics Support Force of the People's Liberation Army of China, Lanzhou 730050, China

## Abstract

异基因造血干细胞移植后合并吉兰-巴雷综合征较为罕见，国内报道很少。吉兰-巴雷综合征发病急，严重威胁患者生命，需尽早诊断及治疗。1例急性髓系白血病患者行异基因造血干细胞移植后5个月余，合并迟发急性肠道移植物抗宿主病后逐渐出现四肢肌无力、眼球运动受限，经脑脊液、肌电图等检查，明确诊断吉兰-巴雷综合征，经大剂量静脉免疫球蛋白治疗，肌力逐渐恢复，预后良好。

## Abstract (en)

Guillain-Barre syndrome rarely develops after allogeneic hematopoietic stem cell transplantation（allo-HSCT）, and only a few reports exist in China. Guillain-Barre syndrome is an acute and life-threatening condition that requires early diagnosis and treatment. A patient with acute myeloid leukemia underwent allogeneic HSCT for >5 months and gradually developed limb muscle weakness and limited eye movement after coexisting with delayed acute intestinal graft-versus-host disease. After the examination of cerebrospinal fluid and electromyography, the diagnosis of Guillain–Barre syndrome was confirmed. After a high-dose intravenous immunoglobulin（IVIg）treatment, muscle strength gradually recovered, and the prognosis was good.

异基因造血干细胞移植（allo-HSCT）患者由于大剂量的预处理、放疗、长期口服免疫抑制剂、合并感染等因素，是神经系统并发症的高危人群，严重影响患者的长期生存[1]–[3]。吉兰-巴雷综合征（guillain-barré syndrome, GBS）又称急性炎症性多根神经病变，是一种累及周围神经和神经根的免疫介导的多发性神经根神经病，常由感染诱发[4]。早期症状具有隐匿性，容易引起漏诊或误诊，造成严重后果。本文报道1例allo-HSCT后并发GBS患者的诊疗过程，为此类疾病的诊治提供参考。

## 病例资料

患者男性，38岁，已婚，因“急性髓系白血病移植后5个月余”于2022年3月入院。患者于2021年1月因头痛、鼻塞1个月余就诊于当地医院，查血常规：WBC 171.3×10^9^/L，HGB 55 g/L，PLT 8×10^9^/L；骨髓象：原幼单核细胞占56％；外周血涂片：原幼单核细胞占85％，免疫分型：67.58％的细胞为恶性髓系幼稚细胞，表达CD117、CD33、CD371，不表达CD16、CD11b、CD7、CD5、CD4、CD8、CD3、CD56、ckappa、lambda、CD19、CD10、CD20、MPO、CD64、CD14、CD42a、TdT、CD22、cCD3、CD15、CD36；粒细胞占有核细胞26.99％；单核细胞占有核细胞2.17％，成熟阶段为主，考虑AML-M4。基因突变：FLT3-TKD、GATA2、NRAS阳性。染色体分裂相较少。综合评估明确诊断AML-M4，经小剂量阿糖胞苷降低肿瘤负荷后给予DA方案化疗，化疗后2周复查骨髓示原始粒细胞46％，考虑未缓解，给予HA方案再次诱导化疗及大剂量阿糖胞苷巩固化疗，于6月给予DA方案，7、8月分别给予中剂量阿糖胞苷化疗，期间复查骨髓象达完全缓解（CR）、微小残留病（MRD）阴性，FLT3-TKD、GATA2、NRAS阴性。于2021年10月行亲缘HLA全相合异基因造血干细胞移植（供者为胞妹，B+供O+），采用改良BuCy预处理方案（阿糖胞苷4 g·m^−2^·d^−1^，−9 d、−8 d；白消安0.8 mg/kg每6 h 1次，−7 d～−5 d；环磷酰胺1.8 g·m^−2^·d^−1^，−3 d、−2 d；司莫司汀250 mg/m^2^，−4 d），共输注单个核细胞5.25×10^8^/kg，CD34^+^细胞9.88×10^6^/kg。以环孢素A、霉酚酸酯、短程甲氨蝶呤联合预防GVHD。同时预防性抗细菌、抗真菌、抗病毒等治疗。移植后出现粒细胞缺乏伴发热及血流感染，先后给予美洛西林钠/舒巴坦钠、亚胺培南-西司他丁钠联合卡泊芬净抗感染治疗，体温逐渐减至正常。+17 d粒细胞、血小板均植活。+28 d复查骨髓未见异常，流式细胞术MRD阴性，嵌合为完全嵌合（NK细胞99.71％，B细胞98.81％，T细胞98.55％），巨细胞病毒（CMV）及EB病毒（EBV）DNA检测均阴性。环孢素A血浓度322.6 µg/L，环孢素A调整为75 mg每日2次。+60 d、+90 d及移植后4、5个月复查血常规正常，骨髓穿刺涂片未见异常，流式细胞术MRD阴性，嵌合为完全嵌合状态，评估病情稳定。2022年3月（移植后5月余）无明显诱因发生腹泻，每日约6～7次黄色水样便，每次约200 ml，每日总量1 200～1 400 ml，伴餐后腹部胀痛，自行口服蒙脱石散效果不佳，遂于3月29日就诊于我科，查体：全身色沉严重，口腔上腭见黏膜白斑。入院查血常规：WBC 5.52×10^9^/L，中性粒细胞4.21×10^9^/L，HGB 158 g/L，PLT 109×10^9^/L；D-二聚体1.56 mg/L、纤维蛋白原降解产物5.40 mg/L、活化部分凝血活酶时间36.2 s；肝功：转氨酶正常，总蛋白46.9 g/L，白蛋白26.9 g/L，球蛋白20.0 g/L，总胆红素34.70 µmol/L，直接胆红素31.10 µmol/L，间接胆红素3.60 µmol/L，γ-谷氨酰基转移酶118.00 IU/L；电解质：钙1.99 mmol/L、钾2.99 mmol/L、钠131.4 mmol/L；尿酸511 µmol/L，估算肾小球滤过率（eGFR）103.33 ml·min^−1^·1.73m^−2^；B型钠尿肽<10.0 ng/L；降钙素原0.286 ng/L，IL-6 10.4 ng/L；G试验及GM试验均阴性；IgG 7180 mg/L，IgA 681 mg/L，IgM 626 mg/L；肺CT：双肺胸膜下多发小结节及斑片影，考虑新发炎症；腹部超声：脂肪肝，脾大（脾门处厚4.3 cm，长径13.4 cm，肋缘下2.0 cm），副脾，胰、双肾声像图未见明显异常；心电图未见异常；骨髓象未见异常，MRD阴性，呈完全嵌合状态。CMV-DNA阴性，EBV-DNA 5.88×10^2^拷贝/ml。考虑迟发急性肠道GVHD（Ⅲ度），给予甲泼尼龙40 mg每12 h 1次静脉滴注及莫西沙星联合阿昔洛韦抗感染治疗，腹泻明显减轻，CT示肺炎好转，此后病情平稳。移植后6个月出现指端麻木，胸四平面躯体深浅感觉减退，肌力Ⅳ级，双侧肢体肌张力稍低，腱反射（+），双侧病理征可疑阳性；于4月16日出现眼球运动受限、口周麻木，查体：患者意识清楚，双侧眼球固定，瞳孔对光反射存在，咽反射弱，双上肢肌力Ⅴ级，双下肢肌力Ⅳ+级，四肢腱反射减弱，四肢共济运动正常，四肢运动觉、震动觉、图形觉正常，全身痛觉查体紊乱，双下肢病理症阳性。颈无抵抗，Kernig征、Brudzinski征均阴性。头颅MRI：双侧半卵圆中心及侧脑室周围白质脱髓鞘改变，双侧基底节区多发扩大V-R间隙，双侧海马沟残余囊肿；眼眶MRI：双侧眼眶MR检查未见明显异常，右侧上颌窦黏膜下小囊肿；胸椎MRI及脊柱平扫未见明显异常；颈椎MRI：颈椎骨质增生，颈椎间盘变性，颈3～4椎间盘膨出，颈4～5椎间盘轻度突出（右旁中央型），颈5～6椎间盘轻度突出（右侧椎间孔型），脊髓形态及信号未见明显异常。双下肢无力逐渐加重，转入神经内科，查体：颈4以下痛觉过敏（双侧膝关节以下平面痛觉过敏较重），双下肢病理症阳性。颈无抵抗，其余肌力查体同前。脑脊液检查：脑脊液蛋白700.0 mg/L（参考值80～430 mg/L）、葡萄糖3.80 mmol/L、氯123.0 mmol/L、钾2.72 mmol/L。脑脊液常规：白细胞计数6×10^6^/L、蛋白定性弱阳性。脑脊液EBV、CMV、水痘-带状疱疹病毒（VZV）、人疱疹病毒6型（HHV-6）均阴性。肌电图示四肢周围性神经损害。患者眼球固定，主要累及动眼神经、滑车神经、外展神经。患者吉兰-巴雷综合症诊断明确，给予静脉注射免疫球蛋白（IVIg）20 g/d ×5 d，甲泼尼龙逐渐减量，双下肢无力较前明显好转，治疗两周后出院。移植后10个月复查原发病病情稳定，患者肌力正常，生活可自理。

## 讨论

GBS的典型临床表现是快速进展的双下肢或上肢无力，严重病例可出现吞咽障碍、呼吸衰竭，可伴有自主神经功能障碍（心动过速、肢体肿胀、尿潴留等）。部分病例可出现不对称的肌无力和感觉异常，或首发症状是不能准确定位的头痛、步态不稳等，容易引起误诊[5]。GBS的诊断主要依据临床表现及脑脊液提示蛋白-细胞分离现象，结合神经电生理检查及影像学检查排除感染、神经根压迫、脑梗死等[6]。GBS的全球年发病率为（0.81～1.91）/10万，高达20％的GBS患者会伴随严重残疾，约5％的患者死亡[7]。关于GBS的具体病因仍不完全清楚，约2/3的GBS患者在症状出现前3～6周有感染史（腹泻、上呼吸道感染等），也有文献报道空肠弯曲菌、肺炎支原体、流感嗜血杆菌、CMV、EBV、戊型肝炎病毒、甲型流感病毒、寨卡病毒与其发病有一定的关系，其致病被认为由细胞免疫和体液免疫共同介导[8]–[9]。GBS的治疗包括IVIg、血浆置换、B族维生素及并发症处理，重症GBS患者需行气管插管及机械辅助通气，必要时进行康复治疗[5],[10]。除了上述治疗，环孢素A在个别病例中可能有效[11]。

国内外学者报道allo-HSCT后GBS的发生率为0.3％～0.8％[12]–[13]。有学者报道了7例移植后GBS，其中6例发生于移植后10 d至12个月[14]，也有报道显示移植后2 d至15个月内均可发生[13],[15]–[16]。

关于移植后并发GBS的原因尚无定论。有报道显示CMV等病毒感染可诱发[14],[17]–[18]，但亦有移植后合并GBS病例中没有病毒感染证据的报道[19]。因此，病毒感染可能只是移植后GBS发生的高危因素。也有文献报道移植后合并GBS与阿糖胞苷等化疗药引起的神经毒性有关，阿糖胞苷的代谢产物阿糖尿苷可引起大脑及小脑功能紊乱并导致GBS[19]。Rodriguez等[20]报道了2例患者行allo-HSCT，预处理方案采用TBI联合阿糖胞苷/环磷酰胺/抗胸腺细胞球蛋白（ATG），均在allo-HSCT后1周内出现手足麻木，双下肢无力逐渐加重，伴呼吸困难，最后确诊GBS。也有报道显示颅脑照射和大剂量甲氨蝶呤等也能使GBS的发生风险增加。

有报道显示移植后合并GVHD可使GBS的发生风险增加[15],[20]。本例患者移植后5个月余出现迟发急性肠道GVHD，最后诊断GBS。Yoshida等[15]报道1例急性白血病患者行allo-HSCT，+86 d出现Ⅲ度急性肠道GVHD，+114 d患者出现四肢远端麻木，伴吞咽困难、呼吸衰竭，经三个疗程IVIg治疗，患者肌力改善，于出现神经症状86 d后脱离辅助呼吸。该患者行腓肠神经活检显示脱髓鞘活跃，巨噬细胞和CD8^+^ T细胞浸润，提示移植后合并GBS的病理特征。

结合以上文献报道，在allo-HSCT前使用大剂量预处理药物如阿糖胞苷或合并急性GVHD，会增加GBS的发生风险。

由于原发恶性肿瘤、移植后免疫力低下、感染、GVHD等因素，相比一般人群，allo-HSCT后合并GBS的治疗效果差，死亡率明显增高[12],[19]。国内学者报道2例allo-HSCT合并GBS患者，虽经IVIg、呼吸机辅助呼吸等积极治疗，但最终均死亡，同时作者统计了国内外共29例移植后合并GBS的患者，最终12例死亡，死亡率高达41.37％[12]。Gonzalez等[20]报道了1例慢性髓性白血病行allo-HSCT，+56 d出现皮肤Ⅱ度急性GVHD，+83 d出现发热、CMV血症，+91 d出现胃肠道出血，考虑胃肠道Ⅱ度急性GVHD，同时逐渐出现震颤和肌肉无力，+92 d出现复视、构音障碍、近端肌无力、呼吸困难，经脑脊液和肌电图检查，诊断GBS，给予血浆置换和大剂量IVIg治疗，但神经系统症状逐渐加重，患者最终死亡，经尸检确诊死于脑弓形虫病与GBS。

本例患者allo-HSCT后5个月余出现肠道急性GVHD，合并双下肢快速进展性无力，脑脊液检查示蛋白-细胞分离，结合神经电生理检查，明确诊断GBS，经大剂量IVIg治疗，肌力逐渐恢复，预后良好。
